# High-throughput sequencing yields a complete mitochondrial genome of the *Cryptotympana atrata* (Insecta: Hemiptera: Cicadidae)

**DOI:** 10.1080/23802359.2021.1934154

**Published:** 2021-06-14

**Authors:** Chenchen Zhao

**Affiliations:** Qufu Normal University Hospital, Qufu Normal University, Qufu, China

**Keywords:** Mitogenome, *Cryptotympana atrata*, high-throughput sequencing, phylogeny

## Abstract

*Cryptotympana atrata* is a common insect pest found in forest ecosystem throughout East and South Asia. In this study, the complete mitochondrial genome of one individual was determined using high-throughput sequencing. The mitogenome is 15,338 bp in length with an A + T content of 77.9%, and contains 13 protein-coding genes (PCGs), 22 transfer RNA genes (tRNAs), two ribosomal RNA genes (rRNAs), and one control region (CR or D-loop). The gene arrangement and composition is similar to other published mitogenomes of Cicadidae. The concatenated PCGs were used to conduct Bayesian phylogenetic analyses together with several related Cicadidae with mitogenome data in GenBank. Phylogenetic analysis shows that two species (*C. atrata* and *C. facialis*) and *Auritibicen bihamatus* were herein corroborated to be the tribe of Cryptotympanini. Our results show the location of genus *Cryptotympana* in Cicadinae and the location of the subfamily in Cicadidae, and provide data for further study of phylogeny in Hemiptera.

Cicadas (Hemiptera, Cicadidae) are well known for their loud calling songs produced by male adults during summer (Young and Bennet-Clark 1995). *Cryptotympana atrata* (Fabricius, 1775), a cicada species, is widely distributed in China, Japan, Korean Peninsula, and North India (Hayashi 1987). *C. atrata* obtain nourishment by sucking the juice from trees and may occasionally cause great damage to their host plants when large numbers of females insert eggs into the stems of trees (Hou et al. 2014). They are seemed as the common insect pests of forest ecosystem. However, little information is available for its agricultural ecology and population genetic diversity due to limited marker resources. Mitogenome is a valuable marker resource and is being widely used for genetics and molecular identification of many Hemiptera. The advancement of high-throughput sequencing technology has facilitated rapid obtainment of mitochondrial genome of animals (Hahn et al. 2013). In the present study, we determined a complete mitochondrial genome of *C. atrata* by using shotgun reads produced high-throughput sequencing.

The *C. atrata* individual was collected from Taian (N36.15°, E117.06°), Shandong Province, China on 10 August 2020. Then, the sample was preserved in the Animal Specimen Museum of Qufu Normal University with the specimen code QF-Ku-Tai-2020023. The person in charge of the collection is Yanning Bai (byn8709@126.com). Total genome DNA was isolated and then sequenced using the Illumina Novaseq sequencing platform (LC-Bio, Hangzhou, China). The short paired-end reads were assembled into contigs by using A5-miseq v20150522 (Coil et al. 2015) and SPAdes v3.9.0 (Bankevich et al. 2012). The gene annotation was performed with MITOS (Bernt et al. 2013). The published mitogenome of *Cryptotympana facialis* (GenBank accession no. MG737718.1) was used as the assembly and annotation references.

The complete mitogenome of *C. atrata* was 15,338 bp in length (GenBank accession no. MW405441) with 77.9% AT and encoded 13 protein-coding genes (PCGs), 22 transfer RNA genes (tRNAs), two ribosomal RNA genes (rRNAs), and a control region (CR or D-loop). All PCGs of *C. atrata* mitogenome have similar locations and strands with that of other published Hemiptera species (Kolokotronis et al. 2016; Liu et al. 2017; Zhong et al. 2019). There is 11,040 bp for the total length of 13 PCGs, which is encoding 3667 amino acids. In the 13 PCGs, the shortest one was ATP8 gene (156 bp) and the longest one was NAD5 gene (1647 bp). Eight PCGs (NAD2, COXI, ATP6, COXIII, NAD4, NAD4L, CYTB, and NAD1) used ATG as start codon, three PCGs (COXII, ATP8, and NAD5) used ATT as start codon, and two PCGs (NAD3 and NAD6) used ATA as start codon. Meanwhile, 10 PCGs (NAD2, COXIII, NAD4, NAD4L, NAD1, COXII, ATP8, NAD5, NAD3, and NAD6) used the typical termination codons TAA, while three PCGs (COXI, ATP6, and CytB) used TAG as termination codons. The 22 tRNA genes size varies from 60 to 70 bp, and the lengths of 12S and 16S rRNA genes are 765 and 1275 bp, respectively.

To assess mitochondrial sequence authenticity of *C. atrata* and its phylogenetic position, the concatenated 13 PCGs of species of family Cicadidae available in GenBank were used to reconstruct the Bayesian phylogenetic tree. The species *Callitettix biformis* (GenBank accession no. JX844627.1) of family Cercopidae was used as outgroup. The phylogenetic analysis showed that *C. atrata* clustered with *C. facialis*, and together formed a monophyletic relationship with *Auritibicen bihamatus* in the tribe of Cryptotympanini (Figure 1), which is consistent with recent molecular studies (Marshall et al. 2018). Taken together, the complete mitogenome of *C. atrata* characterized here should contribute to a better understanding of phylogenetic relationships among Cicadidae species and also serve molecular identification, population genetic and evolutionary biological studies of *C. atrata*.

**Figure 1.**
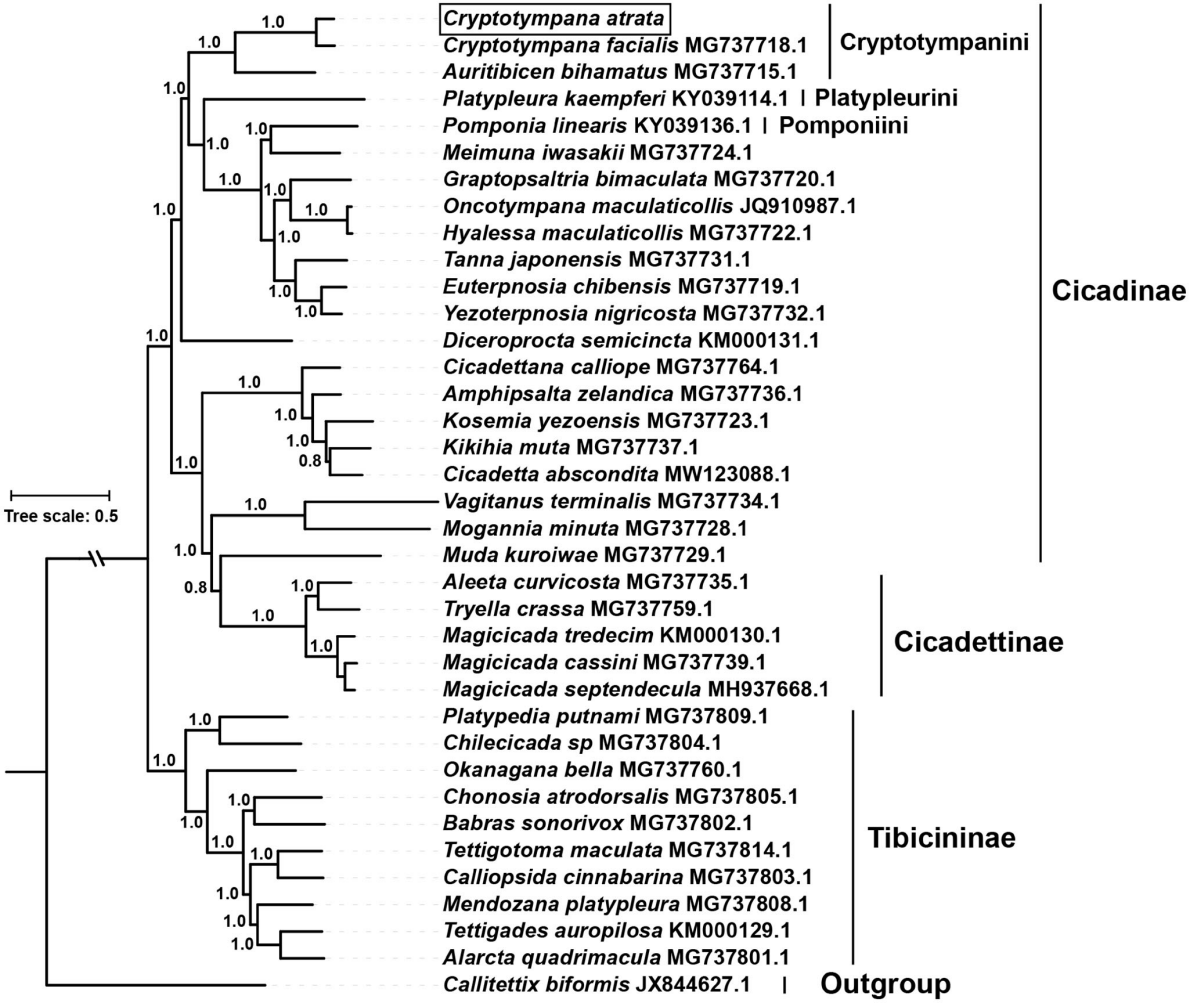
The phylogenetic tree inferred from Bayesian inference using MrBayes v.3.2.7 (Altekar et al. 2004) under GTR + I+G model, based on the concatenated 13 PCGs of 36 species of family Cicadidae and one outgroup. The novel sequencing mitogenome of *C. atrata* is highlighted with a box. GenBank accession numbers are given with species names. DNA sequences were aligned in muscle v3.8.31 (Edgar 2004). The numbers at branches indicate the Bayesian posterior probabilities. The corresponding generations of Bayesian inference when the standard deviation of split frequencies fell <0.01 was 41,000.

## Data Availability

The raw data for *C. atrata* has been deposited in the National Center for Biotechnology Information (NCBI database) SRA: BioProject ID PRJNA704537 at https://dataview.ncbi.nlm.nih.gov/object/SRR13775433. The assembled mitogenome of *C. atrata* in this study are openly available in the NCBI at https://www.ncbi.nlm.nih.gov/nucleotide/, reference number: MW405441.
